# A Randomized Controlled Trial Comparing Er:YAG Laser and Rotary Bur in the Excavation of Caries - Patients' Experiences and the Quality of Composite Restoration

**DOI:** 10.2174/1874210601812010443

**Published:** 2018-05-31

**Authors:** Roxana Sarmadi, Elin Viktoria Andersson, Peter Lingström, Pia Gabre

**Affiliations:** 1 Department of Paediatric Dentistry, Public Dental Health, Uppsala County Council, Uppsala, Sweden; 2 Department of Preventive Dentistry, Public Dental Health, Uppsala County Council, Uppsala, Sweden; 3 Department of Cariology, Institute of Odontology, The Sahlgrenska Academy, University of Gothenburg, Gothenburg, Sweden

**Keywords:** Clinical trial, Composite restorations, Er:YAG laser, Patients’ experiences, Rotary bur, Dental caries

## Abstract

**Objective:**

The aim of this study was to evaluate patients´ experiences of two excavation methods, Er:YAG laser and rotary bur and time required by the methods as well as objective assessments of quality and durability of restorations over a two-year period.

**Methods:**

A prospective, single-blind, randomized and controlled investigation was performed. Patients aged 15 to 40 years with at least two primary caries lesions, which had been radiographically assessed as of the same size, were recruited. In each patient, one cavity was excavated using rotary bur and one using Er:YAG laser technique. The time required for excavations and, where applicable, local anaesthesia, was measured during the treatments. Patient experiences were measured using questionnaires. The quality and durability of restorations were assessed over a two-year period in accordance with modified Ryges criteria and radiographs. Twenty-five patients (mean age 22.6 years) participated in the study. In total, 56 cavities were included of which 28 were treated with Er:YAG laser and 28 were treated with a rotary bur.

**Results:**

The patients associated the laser method with less discomfort. The mean time for excavation by laser was three times longer than by rotary bur (13.2 min *vs*. 4.3 min, *P*<0.0001). Over a two-year period, no statistically significant differences with regard to quality or durability could be seen between the restorations associated with the methods.

**Conclusion:**

The Er:YAG laser technique was more time-consuming than the rotary bur. Despite this, the laser technique caused less discomfort and was preferred as an excavation method by patients.

## 1 INTRODUCTION

Dental caries is a multifactorial disease caused by an imbalance between harmful and protective factors [1]. This imbalance also affects the biofilm on the tooth surface, resulting in an increased number of cariogenic bacteria, and leading to demineralization of the tooth surface [2]. Deep caries lesions need to be treated and lost tooth structure needs to be replaced by artificial materials. The goal of the excavation is the selective removal of carious tissue while preserving the healthy tooth structure and vital pulp [3]. Today it is recommended that cavities of moderate depth are excavated to firm dentine and deep cavities to soft dentin. However, for all cavities, it is important to excavate to hard dentine peripherally [3].

A common technique for removing the carious tissue is the use of rotary bur, a method that has a long history and is still used by most clinicians. The technique is fast and simple to use while it has several drawbacks such as the risk of removing healthy tooth substance, vibration and noise, which may lead to discomfort and pain for the patient and subsequently to dental fear [4, 5]. The disadvantages of using the rotary bur have led to a search for alternative methods to excavate caries lesions, including for example plastic and ceramic burs, sono/air abrasion, chemo-mechanical technique, enzymes and lasers [6]. Several lasers such as the erbium plus chromium-doped yttrium-scandium-galium-garnet (Er,Cr:YSGG) with a wavelength of 2780 nm and erbium-doped yttrium-aluminium-garnet (Er:YAG) with a wavelength of 2940 nm and the 9300-nm CO_2_ laser can be used for caries removal and cavity preparation [7].

The Er:YAG laser was introduced in the 1980s. The method causes vaporization of water and increases the internal pressure of dental tissue which leads to explosive destruction of enamel and dentine. The process of explosive ablation is also called thermo-mechanical effect [8, 9]. Er:YAG laser has a great potential for hard-tissue ablation due to its high absorbability in water and hydroxyapatite and has been shown to remove enamel and dentin using a pulsed laser beam combined with water spray, without noticeable pulp temperature increase [10, 11]. Several studies have also shown that patients prefer the use of the laser to the rotary bur [4, 12, 13] and particularly children have been found to have a greater acceptance of this technology [14]. However, the time required by the laser technique has been reported to require two to three times longer for the caries-removal procedure [15, 16].

The longevity and quality of restorations are important and can be influenced by several factors such as tooth type and location of lesion, material used, as well as factors related to both operator and patient [17, 18]. A systematic review and meta-analysis shows an annual failure rate of 1.8% over a five-year period, with the main reasons for failure of restorations being caries and fractures [19]. Studies of sufficient quality comparing restorations made after excavation of caries lesions using laser technology versus rotary bur are few [20]. Contradictory results regarding micro-leakage and bonding strength between enamel and dentin treated with laser, and restoration materials have been reported [21, 22]. However, studies with a follow up of 6 to 24 months, conclude that caries tissue removal with an Er:YAG laser does not appear to affect the clinical outcome of a restoration [23-25]. Jacobsen *et al* [26] evaluated the use of laser in caries treatment in a systematic review, with a literature search up to January 2009. The conclusion was that laser worked as well as rotary burs when it came to removing caries tissues. However, no conclusions about the effect on the dental pulp, longevity of restorations, children’s view of laser treatment and the cost-effectiveness of the method could be drawn, due to lack of studies of adequate quality.

The aim of this study was to evaluate patients´ experiences of two excavation methods, as well as time requirements and the quality and durability of restorations over a two-year period when carious tissue was removed both with Er:YAG laser and rotary burs. The hypothesis was that a higher number of patients would prefer laser, but that the quality of the restorations would remain the same irrespective of excavation method used.

## 2 MATERIALS AND METHODS

The study was approved by the Ethics Committee at the Faculty of Medicine, Uppsala University (No. 2010/200) and the Radiation Safety Committee in Uppsala County (D10/16). It has also been registered in Clinicaltrials.gov (NCT03080649). All subjects received verbal and written information about the study and informed consent was obtained from all participants before the study started. For participants younger than 18 years old, information was also given to, and consent obtained from, their legal guardians.

### 2.1 Study Design

The study was performed as a prospective, single-blind randomized and controlled investigation using a split-mouth design. A total of 25 patients aged 15 to 37 years old were recruited. They were selected among patients attending the Public Dental Service (PDS) in Uppsala County and identified by their dental therapist when they came for regular dental consultations.

### 2.2 Subjects

Inclusion criteria for participation were as follows: i) between 15-40 years old, ii) at least two lesions with primary caries estimated as being of equal size in bite-wing radiographs, and in need of treatment, iii) comparable pairs of cavities located on either occlusal or approximal surfaces, and iv) the cavities should involve the outer 2/3 of the dentin. For the individual patient, either occlusal or interproximal lesions were compared.

When the dental therapist identified a patient who met the inclusion criteria, patient data was transferred to the Researcher Responsible for the study (RS), an experienced dentist specialized in paediatric dentistry, who examined the bite-wing radiographs on a data screen (Olorin VistaLine VC 1900, Olorin AB, Kungsbacka, Sweden) and who took the final decision whether the patient met the inclusion criteria or not.

The following exclusion criteria were used: i) patients with severe general diseases (ASA>2; ASA Physical Status Classification System 2017) [27], ii) cognitive or intellectual disabilities, iii) patients who required sedation or general anaesthesia, and iv) teeth with periapical pathology, a root filling or non-vital teeth.

### 2.3 Randomization Procedure

When the patients met the criteria for participation in the study, they received information about the study and informed consent forms were sent by post. After agreeing to participate, the patient signed the form and returned it to the clinic in a pre-paid envelope. For each subject, one cavity was treated using the rotary bur and one with Er:YAG laser technique. The randomization procedure was performed by using 30 envelopes each containing a note with one of two different messages. In each patient, the cavities were randomly allocated to rotary bur or Er:YAG laser group and also the order the methods should be used. The envelopes were identical and a dental nurse or a dentist not involved in the arrangement of envelopes selected a sealed envelope at random for each pair of cavities. After opening the envelope, both the clinician and the patient were told which method would be used for each cavity and the order of treatment.

### 2.4 Treatment Procedure

Three experienced dentists at the PDS in Uppsala County performed all treatments, of which one performed most of the treatments (93%). All dentists received education in the laser technique, including laser biophysics, laser safety and usage of Er:YAG lasers in dentistry. In addition, they all had clinical experience of the method. Before the start of the study, the Responsible Researcher (RS) went through the method of the study to ensure that the dentists used the study protocol in a correct and consistent manner and that the laser settings were made in the same way for all restorations. An independent researcher (RS), performed all evaluations over a two-year period without being involved in the treatment of patients.

The following data were registered before and during the treatments:

- Sensibility of the tooth. The sensibility was tested by Vitality Scanner (Model 2006, Sybron Endo, UK). The answers were dichotomous, i.e. sensibility or no sensibility.
- If the patient desired local anesthesia or not. The patients could choose to receive local anaesthesia before excavation or at any time during the excavation.
- Apical status. An apical radiograph was taken and assessed by the dentist carrying out treatment before the caries excavation. The reason was to exclude the patient from the study if the radiograph showed periapical pathology.
- Times required for local anaesthesia and excavation to hard/firm dentin were measured with a timer and noted separately. Excavation time was defined as the point at which the treatment session using the laser or rotary bur started until the cavity was assessed as free of caries and ready for restoration. If the patient required local anesthesia in the middle of the excavation, the time for anesthesia was registered.

The therapist excavated the carious tissue until hard dentine was reached peripherally and firm dentin pulpally. The hardness of dentin was assessed with a dental probe and based on the subjective judgment of the therapist. For treatment with the laser system, Er:YAG laser (AT Fidelius plus 3, Fotona, Slovenia) with a wavelength of 2940 nm was used, with power settings according to manufacturer’s recommendations (Table **1**). High and low-speed hand pieces were used for the preparation of enamel, dentin and carious tissue excavation in the rotary bur group. The therapists were not allowed to use the rotary bur in the laser group and vice versa. One tooth was treated at each visit and the second tooth was treated approximately one week after the first.

The same filling material (Tetric Evoceram, Ivoclar Vivadent, New York, USA) and bonding material (Clearfil SE bond, Kuraray Dental, New York, USA) were used for all cavities after first being etched using phosphoric acid (Top Dent Etch gel 38%, DAB Dental, Sweden). All materials were handled in accordance with the manufacturer's instructions. A bite-wing radiograph was taken after completion of the filling as base-line registrations for later comparisons.

### 2.5 Follow-up Immediately After, then One Week, Six Months, One Year and Two Years after, Treatment

The patients responded to two questionnaires for each treated tooth, one immediately after the treatment and a second questionnaire one week after treatment. The questions were based on patients´ views and statements in an earlier interview study [4]. In the first questionnaire, the participants estimated the discomfort associated with visiting a dentist in general, and with receiving local anaesthesia, by marks on a VAS scale. The remaining questions were related to patients’ experiences regarding the latest used treatment methods. Participants described the degree of discomfort/pain associated with the treatments and if they would choose the laser method in future by indicating a mark on a VAS scale. In addition, in the questionnaire answered one week after treatment, patients indicated in multiple-choice questions whether they had pain or not and if they used analgesics or required treatment due to postoperative pain.

At the assessments after six, 12 and 24 months the patients again answered a short questionnaire where they expressed how uncomfortable it had been to remove carious tissue with the laser technique or rotary bur, and which method they would choose in the future if it became necessary to treat a tooth. The patients marked their agreement/disagreement on a Visual-Analogue-Scale (VAS).

The main researcher (RS) performed all clinical evaluations over the two-year period. The restorations were assessed according to a protocol in which only the numbers of the teeth and restorations were stated, without revealing the method used. The entire material was blinded until after the 24-month check-up.

The following evaluations were performed for each tooth at each assessment and the results were registered in a protocol:

- Sensitivity of the tooth was tested by a Vitality Scanner (model, 2006, Sybron Endo, UK) and recorded dichotomously, i.e. sensibility or no sensibility was noted.
- One clinical photograph with occlusal view of the restoration was taken with an SLR camera (Nikon AF-S DX Micro-Nikkor 85 mm f/3.5G ED VR, Japan).
- One apical and one bite-wing radiograph were exposed in the assessment at 12 and 24 months. The main researcher assessed the radiographs on a monitor (Olorin VistaLine VC 1900, Olorin AB, Kungsbacka, Sweden) in order to identify any secondary caries or periapical pathology.
- Restorations were assessed for retention, marginal integrity, marginal discoloration and secondary caries according to modified Ryges criteria [28] (Table **2**) after six, 12 and 24 months. At six months’ check-up, the assessment of secondary caries was based on clinical examination, while at 12 and 24 month check-ups, the assessment of secondary caries was based on a combination of clinical and radiograph examination.

### 2.6 Statistical Analyses

The sample size was calculated to indicate that approximately 80% of patients would choose laser treatment in the future compared to the null hypothesis of 50%, based on a previous study [12]. Twenty patients were needed for 80% power and 25 patients for 90%. It was decided to include 25 patients to allow for dropout, which would still keep the power between 80 and 90%.

Statistical comparisons for continuous variables were made using linear and generalized linear mixed models, with random patient effects and fixed period and treatment effects. Analyses of patients' views on the degree of discomfort/pain during treatment also included discomfort during the administration of local anesthesia, and visiting the dentist in general, as covariates. In situations where the assumption of normally distributed residuals was not fulfilled, continuous response variables were transformed using natural logarithms. In this case, the estimates were retransformed and reported as ratios. A statistical comparison for risk of reaching restoration score Charlie on the modified Ryges criteria (*i.e*. coded as Charlie = 1, otherwise = 0) was made using a generalized linear mixed model (using a logit link function), with random patient effects and fixed period and treatment effects. When significant period effects (*i.e*. effect of treatment order) were present in the linear and logistic mixed models, this was reported in the results, otherwise, no significant period effects were seen. A t-test with a null hypothesis of 50 (*i.e*. the middle) on the VAS scale was performed for the question “I choose laser if I need to be treated in the future”. A *P*-value <0.05 from two-sided tests was considered statistically significant. Statistical analyses were performed using SAS 9.4 (SAS Institute Inc., Cary, NC, USA) and R v3.3 (R foundation for Statistical computing, Vienna, Austria).

## 3 RESULTS

A total of 32 patients corresponded to the inclusion criteria and were asked to participate in the study, out of which seven declined participation. In some cases, the reason for declining participation was unknown but several patients declined due to lack of time. Twenty-five patients participated in the study, of which 12 were men and 13 women, with a mean age of 22.6 years (median age 20 years, range 15-37 years). Twenty-two patients had one pair of equivalent cavities each (a total of 44 cavities) and the remaining three subjects had two pairs of equivalent cavities (a total of 12 cavities). Thus, a total of 56 cavities were included, of which 28 were treated with laser and 28 with rotary bur. A total of 52, 50 and 40 restorations were evaluated after six, 12 and 24 months respectively, according to the modified Ryge´s criteria. Fig. (**1**) shows the number of questionnaires and restorations which have been evaluated and the number of dropouts associated with all evaluations. The main reasons for dropouts were lack of time and a long way to travel.

Most of the cavities were approximal lesions (79%) while the remaining 21% were occlusal lesions. In the randomization process, a higher number of distal cavities were allocated to the laser group (n=14) and, consequently, more mesial cavities to the rotary bur group (n=12). Ten of the excavations in the laser group required local anaesthesia, compared with 15 of the excavations in the rotary bur group. Baseline data is shown in Table **3**. All teeth had positive sensibility at six, 12 and 24 months’ check-ups and no periapical pathology could be identified at 12 and 24 months at radiograph examinations.

### 3.1 Time Required

**Fig. (2):**
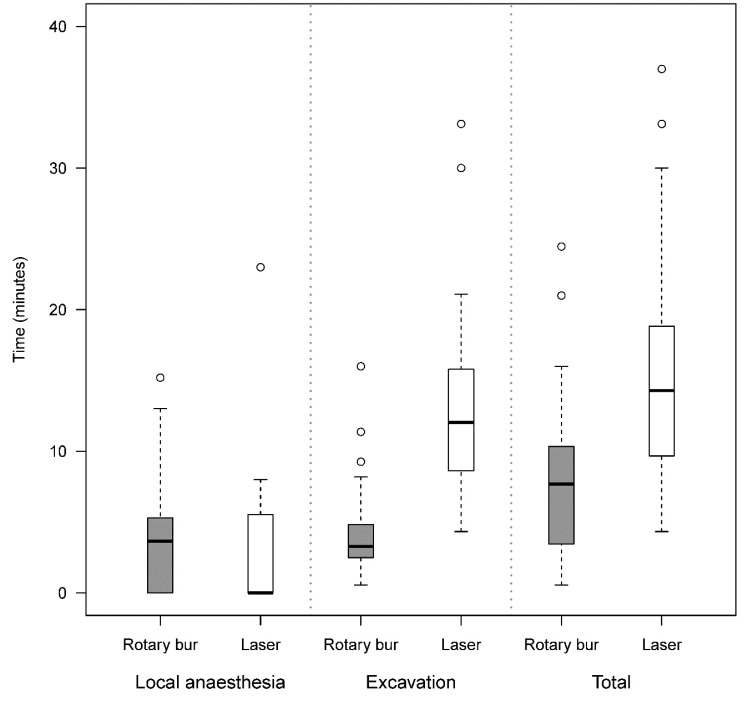
Mean values of time used in minutes for laser and rotary bur methods. The box plot shows the distribution of responses on the five-number summary; minimum, first quartile, median, third quartile and maximum. Statistically significant differences between rotary bur and laser techniques for excavation time and total time (*p*<0.0001).

The mean time for excavation by laser was three times longer than by rotary bur, 13.2 min *vs*. 4.3 min, ratio estimated by mixed model analysis 3.20 (95% CI 2.66-3.85, *p* <0.0001). The mean time for the administration of local anaesthesia in the laser group was shorter than the rotary bur group, because there were fewer patients in the laser group who chose local anaesthesia. The mean total time (anaesthesia and excavation times taken together) was 15.9 minutes for the laser procedure and 8.0 minutes for the rotary bur procedure, ratio estimated by mixed model analysis 2.11 (95% CI 1.68-2.67, *p*<0.0001), (Fig. **2**).

### 3.2 Patients' Views on the Degree of Discomfort/pain During and After Treatment

In general, patients did not feel uncomfortable meeting a dentist (mean value 38.1, 0 not uncomfortable and 100 very uncomfortable in a VAS-scale), while they felt more uncomfortable receiving local anaesthesia (mean value 51.1). Patients indicated marks on a VAS scale directly after treatment and one week afterward. They also gave marks in connection with all evaluations, six, 12 and 24 months after treatment, and described experiences related to the methods used. According to the answers describing the degree of discomfort associated with the treatment, there was no significant difference in discomfort between the methods directly after treatment (estimated mixed model mean difference 0.89, 95% CI -11.59 to 13.37, *p*=0.881). One week after treatment the discomfort was assessed as significantly higher for the rotary bur method (estimated mixed model mean difference 17.39, 95% CI 2.98 to 31.80, *p*=0.021). In the following evaluations, at six, 12 and 24 months, rotary bur treatment was rated with significantly higher discomfort figures than Er:YAG laser (estimated mixed model mean difference at 6 months 30.3 (95% CI 15.9 – 44.7, *p* < 0.001), at 12 months 18.7 (95% CI 5.9 – 31.5, *p* = 0.005) and at 24 months 18.6 (95% CI 4.0 – 33.2, *p* = 0.016). Fig. (**3**) shows a summary of the outcome of this issue. Postoperative symptoms were reported by 26% of the participants after rotary bur treatment and 19% after laser treatment (no statistically significant differences). One participant in the laser group sought dental advice because of pain and one participant in the rotary bur group took analgesics due to pain.

Participants chose the laser method to a significantly higher extent when they were considering statements about their future choice of treatment method, which at all evaluation occasions were in favor of laser treatment. [One week 72.58 (95% CI 60.23-84.94, *p*<0.001 six months 72.9 (95% CI 59.0-86.7, *p*= 0.002), 12 months 71.3 (95% CI 57.3-85.3, *p*=0.005), 24 months 73.5 (95% CI 58.9-88.1, *p*=0.003)].

### 3.3 Restorations

The results of the evaluation of the restorations after six, twelve and twenty-four months are shown in Table **4**. After six months, three of the restorations in the rotary bur group were scored Bravo regarding marginal adaptation, marginal discoloration and secondary caries. After 12 months, two restorations in the rotary bur group were scored Bravo regarding marginal adaptation and discoloration, and two restorations in the laser group were scored Bravo regarding marginal adaptation and secondary caries. Two restorations in each group were scored Charlie regarding secondary caries and needed to be redone. After two years, two additional restorations in the laser group were scored Charlie and were in need of redoing. Consequently, after 24 months four laser-treated cavities (14.8%) had achieved Charlie level, compared to two in the rotary bur treated cavities (7.4%). Although not significant, over 24 months the odds-ratio for reaching the score Charlie in the laser group compared to the rotary bur group was estimated at 2.32 (95% CI 0.33 – 16.19, *p* = 0.38).

## 4 DISCUSSION

This study evaluates patients´ experiences of the excavation method, time requirements and the quality of restorations over a two-year period when carious tissue was removed with Er:YAG laser compared to rotary bur. The results confirmed our initial hypothesis that most patients prefer laser treatment and that the quality of the restorations would be the same irrespective of excavation method, a result in line with previous published studies [4, 12-14, 24, 25].

The study was a randomised controlled single-blind study with each participant acting as his/her own control (split-mouth design), which is a great advantage as it enables within-patient comparisons. The results would have had even greater strength if the study had used a double-blind design. However, this was not possible since it was obvious to the patients which excavation method was used. The single-blind design may have led to patients´ reports of the treatment methods being affected by perceived ideas about the two methods. Another circumstance from the evaluator perspective that could have limited the single-blind method was that cavities created by laser seem to, in radiological appearance, have a more irregular and angular shape compared with cavities created by rotary bur which have a more regular and rounded shape.

The study showed that removal of carious tissue using laser technology took on average three times longer than the rotary bur procedure. The result is in line with other studies showing that caries excavation using the laser method takes two to three times longer than the rotary bur [4, 16]. However, the present study found that fewer patients in the laser group were in need of local anaesthesia which resulted in a total treatment time that was only twice as long as the rotary bur group. This corresponds well with a previous study by Den Besten *et al*. [23] in which fewer patients treated with laser requested anaesthesia than those treated with rotating instruments. Similar results have been reported when comparing chemo-mechanical caries removal with conventional methods [29] and a recently published Cochrane review article has reported the same conclusion [20].

A larger number of distal cavities were randomised to the laser group, which may have partially affected the treatment time for this group as accessing distal cavities could be more time-consuming than treating mesial lesions. A stratified randomization and control of the cavity localization could have been used to achieve an equal distribution. Other possible reasons for the longer excavation time with Er:YAG laser may be that the therapists were less experienced with this method than the rotary bur method. Another possibility is the lack of tactile sensitivity during excavation, which forced the therapist to halt the excavating procedure more often during laser treatment to check the status of the cavity. Regarding tactile sensitivity, the rotary bur has a great advantage based on levels of haptic feedback [7].

The fact that the laser method takes longer is often stated as a disadvantage. However, considering that many patients with dental fear avoid dental treatment because of fear of the rotary bur [4, 5], the laser method enables a treatment that otherwise may not have been conducted. For this group of patients, an opportunity to treat caries lesions before symptoms arise leads to improvement in their oral health, quality of life and self-esteem [30, 31]. However, in this study most of the participants were not frightened of visiting the dentist and yet they still preferred the laser method. The same result has been reported previously in a study by Mosskull Hjertton and Bågesund [16], and this indicates that treatment time is not a very important factor from a patient perspective. This is also in line with our previous interview study where patients expressed willingness to spend more time and pay more if they receive their preferred treatment [4].

Directly after treatment the patients described the degree of discomfort associated with the two methods to be equal, but one week after, the discomfort was significantly higher for the rotary bur method. A similar change of opinion over time has earlier been reported among patients with dental fear. One explanation for the shift in opinion may be that if, prior to treatment, patients´ had preconceived opinions that dental treatment was frightening the fear came back, even if the treatment was carried out successfully [32]. In the same way, the shift in opinion in our study group in favor of laser technique one week after treatment could be explained by a prior positive attitude to laser treatment.

This study shows a cumulative failure rate of 9% for fillings made after excavation with rotary bur and 18.2% after excavation with laser technique over a two-years period, leading to an annual failure rate (AFR) of 4.5% and 9.1% for restorations in rotary bur and laser groups respectively. Opdam et.al showed [19] 1.8% AFR for 2816 posterior composite fillings treated with rotary bur over a 5-years period. They also showed a variation in AFL over a five-years period of between 1.2% to 3.2%, depending on the patients’ degree of caries activity. In this study, we had high-risk caries patients which may be a reason why we got higher AFR than previous studies. The reason why all six fillings needed to be remade was the occurrence of secondary caries. No statistically significant differences between the restorations made using the two methods of excavation were seen, either in quality or survival over a two-year period, even though numerically the survival failure rate of restorations was twice as high for fillings made after laser excavation. However, it cannot be ruled out that a larger number of participants would have probably shown a statistically significant difference in the survival of restorations between the methods. A power calculation based on the primary outcome variable, patients’ experiences, which showed that 25 patients needed to be included, was performed before the study started. A power calculation for the hypothesis that the quality of the restorations should be the same irrespective of excavation method was hard to perform. However, it was planned to include a larger number of participants to get a more stable basis for assessing the fillings, but it turned out to be too difficult to include more patients. In addition, 12 out of 56 restorations could not be evaluated after 24 months due to drop-outs. Although of low scientific value it can be mentioned that eight fillings belonging to four patients who left the study could be reviewed through bitewing radiographs three to four years after the study. No remarks could be noted on radiographs.

## CONCLUSION

In conclusion, this study comparing the use of rotary bur and Er:YAG laser, showed that using lasers took longer, but the method was preferred by the patients. No statistically significant differences between the restorations made after excavation with the two methods could be seen, either in quality or survival of restorations over a two-years period. Longitudinal RCT studies are needed in the future to evaluate the quality and durability of laser restorations over a longer time period.

## Figures and Tables

**Fig. (1):**
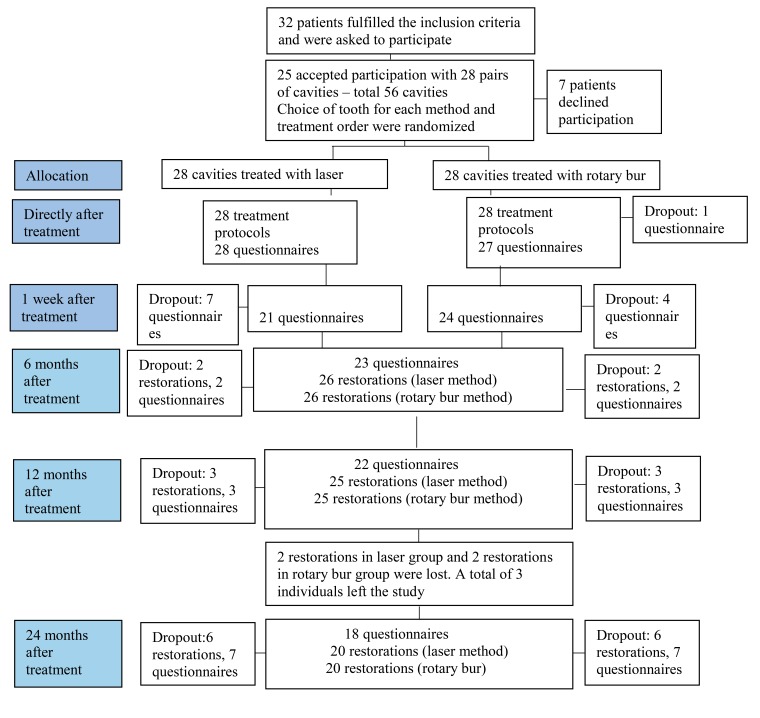
Flow chart of the study from sampling of participants to 24 months after treatment. Immediately after treatment and one week after, one questionnaire was collected per treated tooth. At the evaluation 6, 12 and 24 months after the treatments, one questionnaire was collected per individual and occasion.

**Fig. (3):**
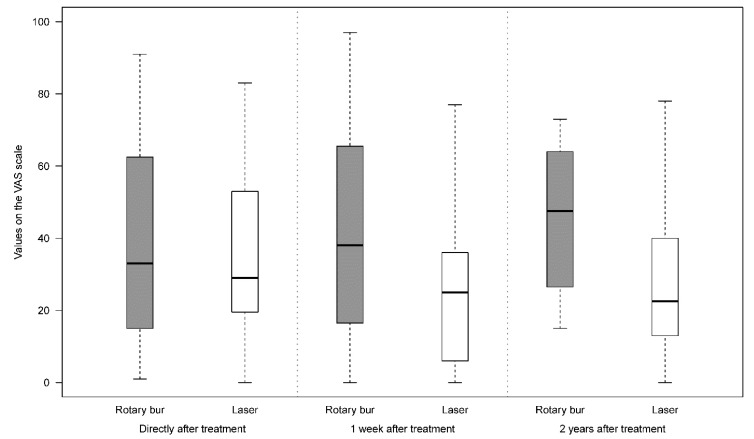
Degree of discomfort/pain directly after, one week and 24 months after treatment. Participants gave a mark from 0 on the VAS scale, indicating no discomfort/pain at all, to 100 to indicate great discomfort/pain. The box plot shows the distribution of responses on the five-number summary; minimum, first quartile, median, third quartile and maximum. Statistically significant differences between rotary bur and laser techniques on all five occasions that were evaluated (*p*<0.001-0.005).

**Table 1:** Recommended settings for preparation of enamel, dentin and excavation of caries with Er:YAG laser (wavelength 2940 nm). The symbol + means that water and air were used.

Indication	Pulse Width	Pulse Energy	Pulse Frequency	Air/Water
Enamel preparation	VSP (SSP)	250-300 mj	30 Hz	+/+
Dentin preparation	VSP/SP	200-300 mj	10-20 Hz	+/+
Excavation dentin caries	VSP/SP	200-300 mj	20-30 Hz	+/+
Excavation deep caries	SP	150-250 mj	5-15 Hz	+/+

**Table 2:** Modified Ryges Criteria.

**Characteristic**	**Evaluation Criteria**
Retention	**Yes**: the restoration is present **No**: the restoration is absent
Marginal adaptation	**Alpha**: no visible signs of ditching along the marginal edge **Bravo**: visible signs of ditching along the marginal edge - small and easy to smooth **Charlie**: slightly deeper ditching, exposed dentine. Treatment is required within a reasonable time **Delta**: restoration is mobile, fractured or partially missing. Treatment is required urgently
Marginal discoloration	**Alpha:** no discoloration **Bravo:** slight discoloration that can be ignored, or that can be polished out **Charlie:** significant edge discoloration that cannot be polished out **Delta:** severe edge discoloration, very pronounced and unfavorable conditions may be suspected, such as micro leakage, caries, or risk of pulp involvement. Treatment required urgently
Secondary caries	**Alpha**: no evidence of caries adjacent to restoration exists **Bravo**: initial caries adjacent to restoration **Charlie**: manifest caries adjacent to restoration. Treatment required urgently

**Table 3:** Baseline data concerning distribution of tooth surfaces and use of local anaesthesia. Number of restorations (proportion) is presented for all variables.

–	**Rotary Bur N=28**	**Laser N=28**
Tooth Surface		
Distal	10 (36%)	14 (50%)
Mesial	12 (43%)	8 (29%)
Occlusal	6 (21%)	6 (21%)
Local anaesthesia	15 (54%)	10 (36%)

**Table 4:** The results of evaluations of restorations after six, 12 and 24 months using modified Ryges criteria.

–	**6 Months**		**12 Months**		**24 Months**	
	Bur	Laser	Bur	Laser	Bur	Laser
	N=26	N=26	N=25	N=25	N=20	N=20
Characteristic	Number (%)	Number (%)	Number (%)	Number (%)	Number (%)	Number (%)
Retention:						
Alpha	26(100)	26(100)	25(100)	25(100)	20(100)	20(100)
Marginal Integrity:						
Alpha	24(92)	26(100)	23(92)	24(96)	19(95)	20(100)
Bravo	2(8)	0	1(4)	1(4)	1(5)	0
Charlie	0	0	1(4)	0	0	0
Marginal Discoloration:						
Alpha	25(96)	26(100)	24(96)	25(100)	20(100)	20(100)
Bravo	1(4)	0	1(4)	0	0	0
Secondary Caries:						
Alpha	24(92)	26(100)	23(92)	22(88)	20(100)	18(90)
Bravo	2(8)	0	0	1(4)	0	0
Charlie	0	0	2(8)	2(8)	0	2(10)
